# Bendamustine for the treatment of relapsed or refractory peripheral T cell lymphomas: A French retrospective multicenter study

**DOI:** 10.18632/oncotarget.10764

**Published:** 2016-07-21

**Authors:** Emilie Reboursiere, Fabien Le Bras, Charles Herbaux, Emmanuel Gyan, Aline Clavert, Franck Morschhauser, Sandra Malak, David Sibon, Florence Broussais, Thorsten Braun, Luc-Matthieu Fornecker, Reda Garidi, Sabine Tricot, Roch Houot, Bertrand Joly, Wajed Abarah, Bachra Choufi, Anne-Dominique Pham, Anne-Claire Gac, Christophe Fruchart, Emilie Marin, Violaine Safar, Anne Parcelier, Hervé Maisonneuve, Emmanuel Bachy, Guillaume Cartron, Arnaud Jaccard, Olivier Tournilhac, Cédric Rossi, Luciane Schirmer, Jean-Alain Martignoles, Philippe Gaulard, Hervé Tilly, Gandhi Damaj, From the Lymphoma Study Association (LYSA) centers

**Affiliations:** ^1^ Department of Hematology, University Hospital of Caen, Caen, France; ^2^ Department of Hematology, Assistance Publique des Hôpitaux de Paris, Créteil, France; ^3^ Department of Hematology, University and Regional Hospital of Lille, Lille, France; ^4^ Department of Hematology, University Hospital of Tours, UMR CNRS, Tours, France; ^5^ Department of Hematology, University Hospital of Angers, Angers, France; ^6^ Department of Hematology, Institut Curie, Paris, France; ^7^ Department of Hematology, Hôpital Necker-Enfants Malades, Paris, France; ^8^ Department of Hematology, Institut Paoli Calmettes, Marseille, France; ^9^ Department of Hematology, Hôpital Avicenne, Bobigny, France; ^10^ Department of Hematology, University Hospital of Strasbourg, Strasbourg, France; ^11^ Department of Hematology, Saint Quentin Hospital, Saint Quentin, France; ^12^ Department of Hematology, Centre Hospitalier Valenciennes, Valenciennes, France; ^13^ Department of Hematology, University Hospital Pontchaillot, Rennes, France; ^14^ Department of Hematology, Centre Hospitalier Sud Francilien, Corbeil, France; ^15^ Department of Hematology, Meaux Hospital, Meaux, France; ^16^ Department of Hematology, Boulogne-sur-Mer Hospital, Boulogne-sur-Mer, France; ^17^ Department of Biostatistics and Clinical Research, University Hospital of Caen, Caen, France; ^18^ Department of Hematology, Hospices Civils de Lyon, Pierre-Benite, France; ^19^ Department of Hematology, University Hospital of Amiens, Amiens, France; ^20^ Department of Hematology, La Roche-sur-Yon Hospital, La Roche sur-Yon, France; ^21^ Department of Hematology, Hospices Civils de Lyon, Pierre-Benite, France; ^22^ Department of Hematology, Université Montpellier, CHRU, UMR CNRS, Montpellier, France; ^23^ Department of Hematology, University Hospital of Limoges, Limoges, France; ^24^ Department of Hematology, University Hospital of Clermont-Ferrand, Clermont Ferrand, France; ^25^ Department of Hematology, University Hospital of Dijon, Dijon, France; ^26^ Department of Hematology, University Hospital of Nancy, Vandoeuvre Les Nancy, France; ^27^ Department of Hematology, Institut de Cancérologie Lucien Neuwirth, Saint-Etienne, France; ^28^ Department of Pathology, University Paris Est, Hôpital Henri Mondor, Créteil, France; ^29^ Department of Hematology, Centre Henri Becquerel, Rouen, France; ^30^ Microenvironnement Cellulaire et Pathologies, Normandie University, UNICAEN, MILPAT, Caen, France

**Keywords:** peripheral T cell lymphoma, bendamustine, efficacy, safety

## Abstract

Peripheral T-cell lymphoma (PTCL) is a group of diseases with poor outcome and few therapeutic options. We aimed to assess the efficacy of bendamustine in real life cohort of patients.

Between November 2009 and March 2015, 138 PTCL patients were treated with bendamustine in 27 centers. Population median age was 64 (28-89) years with male/female ratio of 1.4. There were mainly angio-immunoblastic (AITL = 71), PTCL-not otherwise specified (PTCL-NOS = 40) and anaplastic large cell lymphoma (ALCL = 8). The majority of patients (96%) had disseminated disease and extranodal localizations (77%). Median number of chemotherapy lines prior to bendamustine was 2 (1-8). Median duration of response (DoR) after the last chemotherapy prior to bendamustine was 4.3 months (1-70) and 50% of patients had refractory disease.

Median number of administered bendamustine cycles was 2 (1-8) and 72 patients (52%) received less than 3 mostly because of disease progression. Median dose was 90 (50-150) mg/m². Overall response rate (ORR) was 32.6% with complete response (CR) rate of 24.6% and median DoR was 3.3 months (1-39). AITL patients were more sensitive than PTCL-NOS patients (ORR: 45.1 *versus* 20%, *p* = 0.01). Median PFS and OS were 3.1 (0.2-46.3) and 4.4 (0.2-55.4) months. On multivariate analysis, refractory disease (*p* = 0.001) and extranodal localization (*p* = 0.028) adversely influenced ORR. Grade 3-4 thrombocytopenia, neutropenia and infections were reported in 22, 17 and 23% of cases respectively.

Bendamustine as single agent could be considered as a therapeutic option for relapsed or refractory PTCL, particularly in chemosensitive or AITL patients. Combinations of bendamustine with other drugs warrant further evaluation.

## INTRODUCTION

Peripheral T cell lymphoma (PTCL) is a heterogeneous group of diseases, representing 10-15% of lymphoma. Most PTCL have aggressive forms with poor prognosis [1-3]. Histologic subtypes influences outcome with the best prognosis is attributed to ALK- positive anaplastic-large cell lymphoma (ALCL) [4-7]. The International Prognostic Index (IPI) may be helpful in prognosticating subtypes of PTCL especially ALCL, whereas the Prognosis Index for T-cell lymphoma (PIT) is better discriminant for PTCL-NOS [4, 8]. In the absence of more effective chemotherapy, CHOP (cyclophosphamide-anthracycline-vincristine-prednisone) regimen is the most frequent chemotherapy used in front line. Complete response (CR) rate is about 50% and 5-year overall survival (OS) is 37% [9-13]. Autologous stem cell transplantation (ASCT) as consolidation treatment in first-line showed 5-years OS and progression-free survival (PFS) of 51% and 44%, respectively [14]. Recent results of intensive chemotherapy with upfront autologous stem cell transplantation (ASCT) in eligible patients is promising [15, 16]. Refractory disease or relapses are very frequent, concerning about 70% of patients with no standardized salvage therapy [17, 18]. Cytarabine-based salvage regimens showed an ORR of 63% with CR of 27% and grade 3-4 toxicities between 47 and 61% of cases [19, 20]. Gemcitabine, a nucleoside analog, has shown efficacy as monotherapy, in small cohort of patients, with an ORR of 55% or in combination regimens with oxaliplatin with 30% of CR, but uncommon long-term duration of response (DoR) [21]. In a recently published series describing the population-based experience of the British Columbia Cancer Agency in 153 refractory or relapsed PTCL patients, Mak *et al.* have reported a median OS and PFS of 5.5 and 3.1 months respectively, with no statistically significant difference in outcome after relapse between each of the PTCL subtypes [22]. Pralatrexate, an antifolate, and romidepsin, a histone deacytelase (HDAC) inhibitor, were approved by the Food and Drug Administration for relapsed or refractory PTCL. The ORR and CR rates were 29 and 13% for pralatrexate and 25 and 15% for romidepsin. The median DoR is, however, short with only small subset of patients with long term duration of response under continuous therapy [23, 24]. Brentuximab vedotin, showed, an ORR of 86% in ALCL [25] and 41% in other PTCL subtypes with a CR rate of 24% [26]. Consolidation with ASCT or allogeneic stem cell transplantation (HSCT) in relapse setting for fit patients is the standard of care. However, 2/3^rd^ of patients could not receive transplantation due to disease progression [18, 22, 27, 28].

Bendamustine, a bifunctionnal molecule with alkylating activity and antimetabolites properties has been shown to be effective in a large panel of hematological malignancies [29, 30]. In a recently reported phase II study, 60 patients with PTCL were treated for 6 cycles of 120mg/m^2^ infusions of bendamustine with an ORR of 50% and complete response (CR) rates of 28% [31]. The DoR was 3.5 months with more than one third of patients with a DoR longer than 6 months. Median OS and PFS were 6.2 and 3.6 months, respectively. In a retrospective Italian cohort of 20 PTCL patients, bendamustine demonstrated an ORR of 55%, CR of 10% and 6 months estimated PFS and OS of 44% and 57%, respectively [32]. However, the precise place of bendamustine use among all PTCL treatment strategies is still unclear [33, 34].

In order to assess the efficacy of bendamustine outside clinical trials, we conducted a national retrospective study of patients with the diagnosis of PTCL and who were treated with bendamustine.

## RESULTS

### Patient's characteristics

From November 2009 to March 2015, 138 patients from 27 centers in France treated with bendamustine for a PTCL were analyzed (Table 1). The median age was 64.0 (27.7 to 88.5) years with 22 patients (16%) older than 75 years. The male/female ratio was 1.4 (83/59). Histopathologic subtypes were predominantly angio-immunoblastic T-cell lymphoma (AITL = 71, 51.4%), PTCL not otherwise specified (PTCL-NOS = 40, 29.0%), and ALCL (*n* = 8, 5.8%). The other subtypes were rare including extranodal NK/T cell lymphoma, nasal-type (ENKTCL = 4, 2.9%), advanced-stage mycosis fungoide (MF = 9, 6.5%), EATL (*n* = 2), subcutaneous panniculitis-like-TCL (*n* = 1), hepatosplenic-TCL (*n* = 1) and unclassified PTCL (*n* = 2).

The majority of patients had disseminated-stage disease (*n* = 127; 96.2%), with extranodal localizations (*n* = 99/128; 77.3%) including bone marrow involvement (*n* = 52/124; 41.9%)The most common extranodal localizations outside bone marrow involvement were skin (*n* = 17/138; 12.3%) and lung (*n* = 3/138; 2.2%). The IPI was high in 74.8% of patients (*n* = 101).

The median number of prior lines of chemotherapy was 2 (range 1-8) (Table 1). The most frequent prior chemotherapy used was CHOP/CHOP-like regimens in 122 patients (88.4%) and cytarabine-based regimens in 53 (38.4%) patients. Only 16 patients had autologous stem cells transplantation (ASCT = 11%) and 7 (5%) patients had allogeneic SCT prior to bendamustine. The median DoR of chemo-sensitive patients after the last chemotherapy was 4.3 months (1-70). Sixty-nine (50%) patients had refractory disease when bendamustine treatment was initiated. Median time from diagnosis to bendamustine first infusion was 12.1 months (range 1.5-108.1). Of note, there were no significant difference for main patients' characteristics such as age, disease stage, number of previous line and disease status at bendamustine between AITL and PTCL-NOS patients at study entry (data not shown).

**Table 1:** Patients' demographics and disease characteristics at Bendamustine

Characteristics	*N.*	%
Patients	*138*	
Age, years		
Median (range)	64 (27.7-88.5)	
> 65 years	*62*	43.7
Sex		
Male	*82*	59.4
Female	*56*	40.6
Histology		
AITL	*71*	51.4
PTCL-NOS	*40*	29.0
ALCL	*8*	5.8
NKTCL	*4*	2.9
MF	*9*	6.5
others	*6*	4.4
Ann Arbor Stage	*132*	
I-II	*5*	3.6
III-IV	*127*	96.2
IPI	*135*	
1-2	*34*	25.2
3-5	*101*	74.8
Extra-nodal site involvement	*99/128*	77.3
Bone marrow involvement	*52/124*	41.9
Previous lines of treatment		
Median (range)	*2(1-8)*	
1	*46*	33.3
2	*55*	39.9
3 or more	*37*	26.8
Prior therapy		
ASCT	*16*	11.5
CHOP/CHOP-like regimen	*122*	88.4
Cytarabine-based regimens	*53*	38.4
Others	*11*	8.0
Time from diagnosis to bendamustine, months		
Median (range)	12.1 (1.5-108.1)	
Refractory to last prior therapy	*69*	50.0

Patients' demographics and disease characteristics at Bendamustine Abbreviations: AITL, angioimmunoblastic lymphoma; ALCL, anaplastic large-cell lymphoma; ASCT, autologous stem-cell transplantation; CHOP, cyclophosphamide, doxorubicin, vincristine, and prednisone; IPI, International Prognostic Index; MF, mycosis fungoides; NKTCL, NK/T cell lymphoma; PTCL-NOS, peripheral T cell lymphoma non other specified

### Bendamustine administration schedule

Bendamustine was given in all patients as monotherapy at a median dose of 90mg/m^2^ (40-150). The dosage varied according to patients' age, previous treatments and comorbidities based on physician discretion (Table 2). Fifty-four (39.1%) patients received 120 mg/m² at day 1 and 2. Seventy-two (52.2%) patients received fewer than 3 cycles, mainly due to disease progression (95.8%, 69/72). Overall, they received a median of 2 cycles (1-8). Sixty-six (47.8%) patients received 3 cycles or more and 30 (21.7%) patients received 6 cycles.

**Table 2:** Bendamustine administration schedule

	*N*.	%
Dose		
Median (range)	90.0 (40-150)	
<90 mg/m2	*19*	13.7
≥90 mg/m2	*119*	86.2
Dose reduction	*15*	10.8
Number of cycles		
Median (range)	*2.0 (1-8)*	
<3 cycles	*72*	52.2
≥3 cycles	*66*	47.8

### Efficacy

The best ORR, as per the IWGC, was 32.6% (45/138) with a PR rate of 7.2% (10 patients), and a CR rate of 24.6% (34 patients) (Table 3). The median DoR was 3.3 months (1-39), 3.54 months for CR patients and 3.18 months for PR patients (*p* = 0.45). One third (31%) of responders had durable response for more than 6 months. Six patients with PR after 3 cycles converted their response to CR after 6 cycles (6/18; 33.3%).

**Figure 1:**
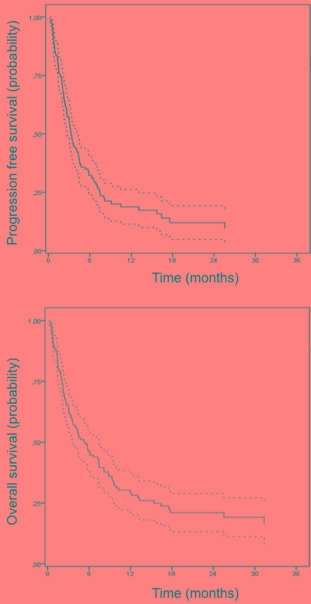
Progression-free survival (PFS) and Overall survival (OS) in the intent-to-treat population (n=129)

Median PFS was 3.1 months (range 0.2-46.3) and median OS was 4.4 months (range 0.2-55.4) (Figure 1). Of note, 9 patients (6.5%) received allogeneic SCT in CR.

For the 54 (39.1%) patients who received the dose of 120mg/m^2^, the ORR was 37% (*n* = 20/54) including 31.4% of CR (*n* = 17). The median DoR and PFS were 3.5 months and 3.9 months, respectively.

Patients older than 75 years represent 16% of the all cohort (*n* = 22) with an ORR of 50% including 37.5% of CR. The median DoR was 5.9 months (1-28.5).

Responses according to PTCL subtypes were different. ORR and CR were respectively of 45.1% (32/71) with 35.2% of CR for AITL patients, whereas it was 20.0% (8/40) with 15% of CR for patients with PTCL-NOS (*p* = 0.01) (Table 3-4). For AITL patients, the median DoR was 3.3 months (1-35.5) and median PFS was 3.6 months (0.2-41.7) with no difference with PTCL-NOS patients (Table 3).

**Table 3:** Response to Bendamustine

	AITL *N = 71*	PTCL-NOS *N = 40*	Total *N = 138*
Overall response rate at the end of treatment *N.* (%)			
ORR	*32* (45.1)	*8* (20.0)	*45* (32.6)
CR	*25* (35.2)	*6* (15.0)	*34* (24.6)
PR	*7* (9.9)	*2* (5.0)	*10* (7.2)
Stable	*0* (0.0)	*2* (5.0)	*3* (2.2)
Progressive	*39* (54.9)	*32* (80.0)	*90* (65.2)
Median time from bendamustine to response, months (_95_CI)	3.3 (0.9-11.1)	3.4 (1.0-7.7)	3.1 (0.4-11.1)
Median DoR, months (_95_CI)	3.3 (1.0-35.5)	3.2 (1.0-38.8)	3.3 (1.0-38.8)
Median OS, months (_95_CI)	4.5 (0.2-55.4)	4.4 (0.7-46.3)	4.4 (0.2-55.4)

Abbreviations: AITL, angioimmunoblastic lymphoma; CR: complete response; DOR: Duration of response; OS: overall survival; OS: overall survival; PFS: progression free survival; PR: partial response; PTCL-NOS, peripheral T cell lymphoma non-otherwise specified.

### Prognostic factors

In univariate analysis, ORR was affected by age (*p* = 0.007), sex (*p* = 0.025), PTCL subtype (PTCL-NOS *versus* AITL) (*p* = 0.01), extranodal localizations (*p* = 0.036) and disease status (refractory *versus* relapsed) at bendamustine (*p* < 0.001) (Table 4).

In multivariate analysis, disease status [OR .1; CI(0.0-04); *p* = 0.001] and extranodal disease [OR 0.2; CI(0.0-0.8); *p* = 0.028] at bendamustine initiation remained the only factors that negatively influenced the ORR (Table 5).

**Figure 2:**
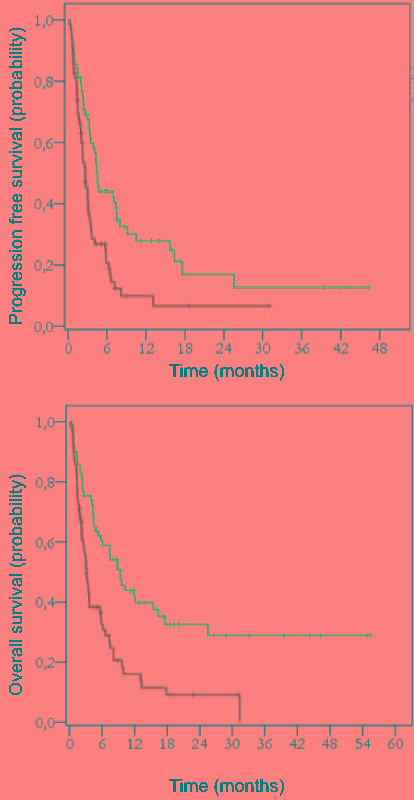
Progression-free survival (PFS) (p=0.001) and Overall survival (OS) (p<0.001) according to chemotherapy status at the bendamustine initiation in the intent-to-treat population (n=129) Blue curve: chemosensitive patients at bendamustine institution Green curve: chemo-refractory patients at bendamustine institution

Overall survival was negatively impacted by the number of previous chemotherapy lines [HR 1.85; CI(1.17-2.93); *p* = 0.008], disease status at bendamustine [HR 5.61; CI(2.33-13.53); *p* < 0.001] and IPI [HR 3.19; CI(1.34-7.59); *p* = 0.009]. In the same way, the number of prior treatment [HR 1.77; CI(1.18-2.67); *p* = 0.006], disease status [HR 3.28; CI(2.77-3.79); *p* = 0.001] and IPI [HR 3.13; CI(1.41-6.96); *p* = 0.005] influenced PFS (Table 5 and Figure 2).

**Table 4:** ORR and PFS Analysis According to Key Subsets (univariate analysis)

Characteristics	*N*.	OR	ORR*_95_CI	*p*	HR	PFS†_95_CI	*p*
Age							
< 65 years	*76*	1			1		
≥ 65 years	*62*	2.75	(1.3-5.7)	*0.007*	0.91	(0.62-1.3)	*0.640*
Sex							
Male	*82*	1			1		
Female	*56*	2.28	(1.1-4.7)	*0.025*	0.51	(0.34-0.8)	*0.001*
Histology							
AITL	*71*	1			1		
PTCL-NOS	*40*	0.2	(0.1-0.5)	*0.010*	1.69	(1.08-2.6)	*0.040*
Ann Arbor stage							
I-II	*5*	1			1		
III-IV	*127*	2.00	(0.2-18.4)	*0.541*	1.22	(0.49-3.0)	*0.663*
IPI							
1-2	*34*	1			1		
3-5	*101*	1.47	(0.6-3.5)	*0.381*	1.47	(0.93-2.3)	*0.097*
Extra-nodal site involvement							
No	*29*	1			1		
Yes	*99*	0.42	(0.2-0.9)	*0.036*	1.31	(0.84-2.0)	*0.238*
Bone marrow involvement							
No	*72*	1			1		
Yes	*52*	0.52	(0.2-1.2)	*0.119*	1.47	(0.94-2.3)	*0.090*
Previous lines of treatment							
1	*46*	1			1		
2 or +	*92*	0.49	(0.2-1.0)	*0.059*	1.68	(1.12-2.5)	*0.013*
Status at bendamustine							
Sensitive	*69*	1			1		
Refractory	*69*	0.17	(0.1-0.4)	*<0.001*	1.89	(1.28-2.8)	*0.001*

Abbreviations: AILT, angioimmunoblastic lymphoma; HR, hazard ratio; IPI, International Prognostic Index; N, number of patients; OR, odds ratio; ORR, overall response rate; PFS, progression-free survival; PTCL-NOS peripheral T-cell lymphoma non other specified

* Logistic regression.

† Cox regression.

**Table 5:** ORR and PFS Analysis According to Key Subsets (multivariate analysis)

Characteristics	OR_ajusted_	ORR_95_CI	*p*	HR_ajusted_	PFS_95_CI	*p*	HR_ajusted_	OS_95_CI	*p*
IPI									
1-2				1	(1.41-3.48)		1	(1.34-7.59)	
3-5				1.45		*0.050*	3.19		*0.009*
Extra-nodal site involvement									
No	1	(0.0-0.8)							
Yes	0.2		*0.028*						
Previous lines of treatment									
0, 1				1	(1.18-2.67)		1	(1.17-2.93)	
2 or +				1.77		*0.006*	1.85		*0.008*
Status at bendamustine									
Sensitive	1	(0.0-0.4)		1	(2.77-3.79)		1	(2.33-13.53)	
Refractory	0.10		*0.001*	3.28		*0.001*	5.61		*<0.001*

### Safety

After a median follow up 4.4 months, 72% of patients (99/138) died. The causes of death were: disease progression (93.9%, *n* = 94) or toxicities (5.0%, *n* = 5). Grade ¾ thrombocytopenia, neutropenia and infections occurred in 22.4%, 16.7% and 22.5% of cases, respectively (Table 6). The main grade 3-4 infections included bacterial sepsis, septic shock, Clostridium colitis and pneumonia. The five toxic deaths were secondary to septic shock with clostridium colitis (*n* = 1), pneumonia with pseudomonas aeruginosa (*n* = 1), toxicity related mortality post allogeneic stem cell transplantation (*n* = 2) and pneumocystis jiroveci pneumonia (*n* = 1).

**Table 6:** Grade 3 to 4 adverse events in patients

Adverse event	*N*.	%
Total events	*94*	60.9
Thrombocytopenia	*31*	22.4
Neutropenia	*23*	16.7
Infections*	*31*	22.5
Others†	*7*	5.0

* Infections include bacterial sepsis, septic shock, Clostridium colitis, pneumonia, Pneumocystis jiroveci pneumonia (n=1)

† Others include cardiac arrhythmia (n=1), anaphylactic shock (n=1), hemolytic anemia (n=1), hepatitis (n=1), skin rash (n=1), venous thrombosis (n=1) and myelodysplasia (n=1; after 18 months of 8 Bendamustine courses).

## DISCUSSION

This retrospective study confirms the efficacy of bendamustine in a large cohort of PTCL patients treated outside clinical trials with a CR rate of 24.6%. These results are concordant with the two prior studies, the prospective BENTLY trial [31] and the retrospective Italian study [32]. However, the ORR (32.6%) and PR rate (7.2%) rate are lower in this cohort than in the prospective BENTLY study (ORR = 50%; PR = 26%). In this study, patients were more intensely treated before bendamustine with an increased number of patients with more than 2 lines of previous chemotherapy, lower dose (90 mg/m²) and less cycles of bendamustine than in the BENTLY study. The low percentage of partial responders as compared to the previous studies is difficult to explain. However, we could hypothesize that lower stringent criteria were applied to stop the treatment in this cohort of highly aggressive disease than in a prospective study. We emphasize that one third of patients in PR after 3 cycles converted their response to CR at 6 cycles, which may indicate an advantage to pursue the treatment if any response is reached.

Patients' other characteristics in this cohort are similar to those reported in the two previous ones regarding age, sex ratio and disease stage. However, the high frequency of AITL in the current cohort is in keeping with the recent report of the high prevalence of AITL in France [35].

This study can be helpful to predict patients who are more likely to respond to bendamustine. Patients with AITL were more sensitive to this drug than patients with other pathological subtypes (univariate analyses) as has been suggested previously [31]. We found a good prognosis impact of having chemosensitive disease status without extranodal localization on response and outcome. We showed also an improved outcome if patients had lower than 3 prior therapies lines or IPI lower than 3 (multivariate analysis).

Bendamustine is effective and safe, even in elderly patients, older than 75 years. ORR and CR rates were not different from those of younger patients. This efficacy and the toxicity profiles of the drug could suggest its preferential use in this group of patients aged more than 70 years old. Furthermore, it could be used as a bridge to transplant in younger patients.

In conclusion, bendamustine may represent an alternative therapy for relapsed or refractory PTCL patients in real-life settings and could be considered as a salvage strategy. These results could be helpful to select patients who will be more likely to respond to bendamustine. Having chemosensitive disease at relapse after being treated with less than 3 lines of chemotherapy are predictive factors for response. The DoR is unfortunately short as with other multiple single agent treatment suggesting the need for evaluating combination drugs in prospective trials.

## MATERIALS AND METHODS

Hematological French centers were asked to report retrospectively the results of the use of bendamustine in refractory or relapsed PTCL patients. Patients aged 18 years old or more with the diagnosis of PTCL were included. Primary cutaneous T_cell lymphoma with a stage less than IIB [36], Sezary syndrome, the leukemic forms according to the WHO classification [3] and patients who received bendamustine in the BENTLY trial [31] were excluded from the analysis. Patients' demographics and clinical characteristics, histologic subtypes, prior therapies, disease status, bendamustine dosage and schedule were reported. Pathological review through Lymphopath was available for 80 (58%) patients [35]. Responses were evaluated according to the International Working group criteria (IWGC) [37] and International Society for Cutaneous Lymphomas/European Organization for Research and Treatment of Cancer (ISCL/EORTC) revision classification [36]. Toxicities were assessed according to the adverse events recording using the National Cancer Institute's Common terminology Criteria for Adverse Events (NCI CTCAE) version 4. PFS and OS distribution were calculated using Kaplan-Meier estimates. Patients who underwent allogeneic stem cell transplant after bendamustine treatment were excluded from the analyses of the DoR, OS and PFS. PTCL-NOS and AITL comparison was assessed by Chi-2 or Fisher's exact tests for qualitative variable and Mann-Whitney test for quantitative variable. Predictive variables for ORR were determined by using uni- and multivariate logistic regression. Results were expressed as odds ratios and confidence intervals (_95_CI). For OS and PFS, we used the Cox proportional hazards models with a stepwise backward variable selection approach (p≤0.20) for multivariate analysis and to obtain hazard ratios with confidence intervals. All reported p values were two-sided, and the significance limit was set at 5%.
